# The Advances of Laparoscopic Gastrectomy for Gastric Cancer

**DOI:** 10.1155/2017/9278469

**Published:** 2017-09-05

**Authors:** Yeon-Ju Huh, Joo-Ho Lee

**Affiliations:** Department of Surgery, Ewha Womans University Mokdong Hospital, Yangcheon-gu, Seoul 07985, Republic of Korea

## Abstract

Laparoscopic gastrectomy is evolving. With the increasing expertise and experience of oncologic surgeons in the minimally invasive surgery for gastric cancer, the indication for laparoscopic gastrectomy is expanding to advanced cases. Many studies have demonstrated the benefits of minimally invasive surgery, including reduced risk of surgery-related injury, reduced blood loss, less pain, and earlier recovery. In order to establish concrete evidence for the suitability of minimal invasive surgery for gastric cancer, many multicenter RCTs, comparing the short- and long-term outcomes of laparoscopic versus open surgery, are in progress. Advances in laparoscopic gastrectomy are moving toward increasingly minimally invasive approaches that enable the improvement of the quality of life of patients, without compromising on oncologic safety.

## 1. Introduction

In spite of a decrease in its incidence, gastric cancer is still the fifth most common malignancy and the third leading cause of cancer-related death in the world (723,000 deaths, 8.8% of the total) according to GLOBOCAN, 2012. Half of the total global incidence occurs in Eastern Asia (mainly in China) [1]. Especially in Korea and Japan, gastric cancer is one of the most prevalent malignancies, and the proportion of early gastric cancer (EGC) has increased, partly because of nationwide surveillance [2, 3]. Given the heightened incidence of early-stage gastric cancer, the continuing accumulation of surgical experience, and the concomitant advances in instrumentation, the laparoscopic approach has become more commonly employed for the treatment of gastric cancer. Since the 1990s, laparoscopic surgery has been performed for the treatment of EGC in patients with a relatively low risk of lymph node metastasis. Here, the meaningful surgeries that have been historical turning points were summarized (Table 1). Ohgami et al. [4] reported a laparoscopic wedge resection using a lesion-lifting method in 1991, which was the first case of laparoscopic surgery for stomach cancer. Intragastric mucosal resection was also reported by Ohashi [5] in the early 1990s. In 1994, successful laparoscopy-assisted distal gastrectomy (LADG) with lymph node dissection (LND) for EGC was introduced by Kitano et al. [6]. With the development of endoscopic treatments, such as endoscopic mucosal resection and endoscopic submucosal dissection, the need of laparoscopic wedge resection and intragastric mucosal resection has decreased, whereas the application of LADG with LND has increased for treating patients with EGC who are at potential risk of lymph node metastasis. Laparoscopic surgery for EGC has become popular based on several prospective randomized controlled trials (RCTs) that generally reported improved short-term surgical outcomes with comparable oncological safety to that of open surgery [7–10].

## 2. Advances in the Technical Aspect

### 2.1. Hand-Assisted Laparoscopic Surgery (HALS)

The technical difficulties of laparoscopic surgery include the limited mobility of the instruments and the absence of tactile sensation. In HALS, however, the surgeon's left hand is inserted into the abdominal cavity through a special pressurized sleeve, approximately 6-7 cm long, which preserves the operator's tactile sensation [11]. Incorporating both the laparoscopy-assisted open surgical field and the high resolution of laparoscopy, HALS combines the advantages of laparoscopic surgery and laparotomy. Increased tactile sensation and depth perception with the inserted hand might solve several of the technical difficulties of gastrectomy with LND [11]. In the early phase of the development of laparoscopic surgery, it also conferred the advantages of relatively low morbidity and high operative safety. Kim et al. [12] suggested that hand-assisted gastrectomy could be a good learning technique for laparoscopic gastrectomy beginners. However, there are some disadvantages to this method. A hand can encroach upon the intra-abdominal working space, and the sleeve for maintaining the pneumoperitoneum is expensive. Moreover, HALS is ergonomically unfavorable for surgeons, which leads to shoulder and forearm fatigue and strain for the surgeon [13]. As surgical expertise has evolved over time, the number of surgeons who use this method has definitively decreased.

### 2.2. Laparoscopy-Assisted Gastrectomy

In laparoscopy-assisted gastrectomy (LAG), after laparoscopic full mobilization of the stomach, resection of the stomach and anastomosis is performed extracorporeally through a small incision in the epigastrium for the accurate localization of the lesion and secure reconstruction. For the precise localization of the lesion in LAG, the effectiveness of endoscopic clips and laparoscopic ultrasonography has been reported [14]. The usefulness of intraoperative navigation, using three-dimensional (3-D) computed tomographic (CT) angiography during extended LND in LADG, was also emphasized [15], as was the importance of the preoperative identification of vascular trees by 3-D CT angiography [16].

Laparoscopy-assisted total gastrectomy (LATG) is technically demanding in terms of reconstruction and LND such as stations 11d, 4sa, and 10 [3, 17]. Therefore, the use of LATG for the treatment of upper gastric cancer has not been generalized. Various reconstruction methods for esophagojejunostomy have been reported, using circular- and linear-stapled anastomosis.

### 2.3. Totally Laparoscopic Gastrectomy

In LAG, the surgical process via minilaparotomy is sometimes difficult to perform, especially in patients with obesity [18]. Totally, laparoscopic distal gastrectomy (TLDG) that all procedures are carried out laparoscopically has been made possible pursuing minimal invasiveness. TLDG received attention when the first delta-shaped anastomosis was introduced [19]. Several intracorporeal anastomosis techniques have been reported, including Billroth II anastomosis using linear staplers, beta-shaped Roux-en-Y reconstruction, overlap method, and semi-loop after total gastrectomy and inverted T-shaped anastomosis using linear staplers [20–24]. Nowadays, the totally laparoscopic procedure is performed for advanced cancer or remnant cases. The first case of totally laparoscopic total gastrectomy for completion was reported by Shinohara et al. [25].

### 2.4. Reconstruction Methods

For the standardization of laparoscopic gastrectomy, the procedure must achieve reproducibility, safety, and simplicity in terms of not only the LND but also the reconstruction component. Billroth I anastomosis has been known to offer the advantages of greater simplicity of performance, fewer postanastomosis anatomical changes, increased physiological pathways, and lower incidences of adhesion and internal herniation. Consequently, it has been the most commonly performed anastomosis after distal gastrectomy in Korea and Japan [26]. During laparoscopic surgery, intracorporeal gastroduodenostomy has been typically considered a challenging technique because of the difficulty of operating in the narrow working space around the duodenal stump. Therefore, the laparoscopy-assisted reconstruction procedure has been popularly performed for a long time.

#### 2.4.1. Delta Anastomosis

Recently, delta-shaped anastomosis, one of the well-established intracorporeal gastroduodenostomy methods, is popularly performed; however, it still demands delicate and precise laparoscopic techniques of the operators and assistants. The technique proceeds in the following way. First, the duodenal bulb is transected using an endoscopic linear stapler at a 90° angle from the usual line. The stomach is then divided in the customary fashion. Small entry holes are created along the edge of the stomach and the duodenum. The posterior walls of both the stomach and the duodenum are approximated using a 45 mm linear stapler. Then, the staple line is meticulously inspected for any defects to ascertain the color of the anastomosis, after which the common entry hole is closed with one or two linear staplers [27]. The most serious problem regarding anastomosis is leakage. Poor blood supply and excessive tension at the anastomotic site may cause the leakage of anastomosis. Regarding blood supply, the delta technique, in particular, carries a potential risk of anastomosis ischemia since the tissue around the duodenal stump has to be dissected to prepare a sufficient space for the anastomosis. In Billroth I reconstruction, the small size of the remnant stomach might lead to anastomotic-site tension and leakage [28]. Therefore, in case of small remnant stomach or short duodenal bulb, a Billroth II or Roux-en-Y reconstruction might achieve a safer anastomosis.

#### 2.4.2. Roux-en-Y Anastomosis

The Roux-en-Y method is known to be favorable to postoperative quality of life (QOL) with lower incidence of bile reflux and anastomotic leakage [10, 11]. However, it is more laborious because it requires the creation of two anastomoses and one duodenal stump closure. The jejunum is divided at a point approximately 15 cm distal to the ligament of Treitz and jejunojejunostomy which is performed 30–40 cm distal from the jejunal division using a linear stapler. After the creation of the jejunojejunostomy, side-to-side anastomosis between the distal part of the divided jejunum and remnant stomach is carried out by using a linear stapler. The common entry hole is closed by hand-sewn running suture or using linear stapler [29]. Anastomotic leakage hardly occurs after Roux-Y gastrojejunostomy because of the good blood supply and minimal tension at the anastomosis site. Meanwhile, there are some reports that an excessively long Roux-limb can cause internal herniation or Roux stasis syndrome [29, 30]. In addition, even though reflux is known to be rare in the RY group, there is debate remaining as to whether the incidence of reflux-related gastritis is different or not. Therefore, apparent superiority of any particular method was not confirmed. Extracorporeal jejunojejunostomy via periumbilical minilaparotomy is generally accepted. The intracorporeal anastomosis is time-consuming and technically demanding in the hands of unskilled and inexperienced surgeons, and for the extraction of the resected stomach, an incision of at least 3 cm is necessary anyway. Therefore, some surgeons prefer making extracorporeal jejunojejunostomy through periumbilical small incisions in order to reduce operating time.

#### 2.4.3. Esophagojejunostomy Using Circular-Stapled Anastomosis

Owing to the difficulty of achieving a secure purse-string suture and the technical challenge of performing an esophagojejunostomy in a narrow and deep surgical field, LATG or laparoscopic total gastrectomy (LTG) is performed relatively less often compared with LADG.

**Figure 1:**
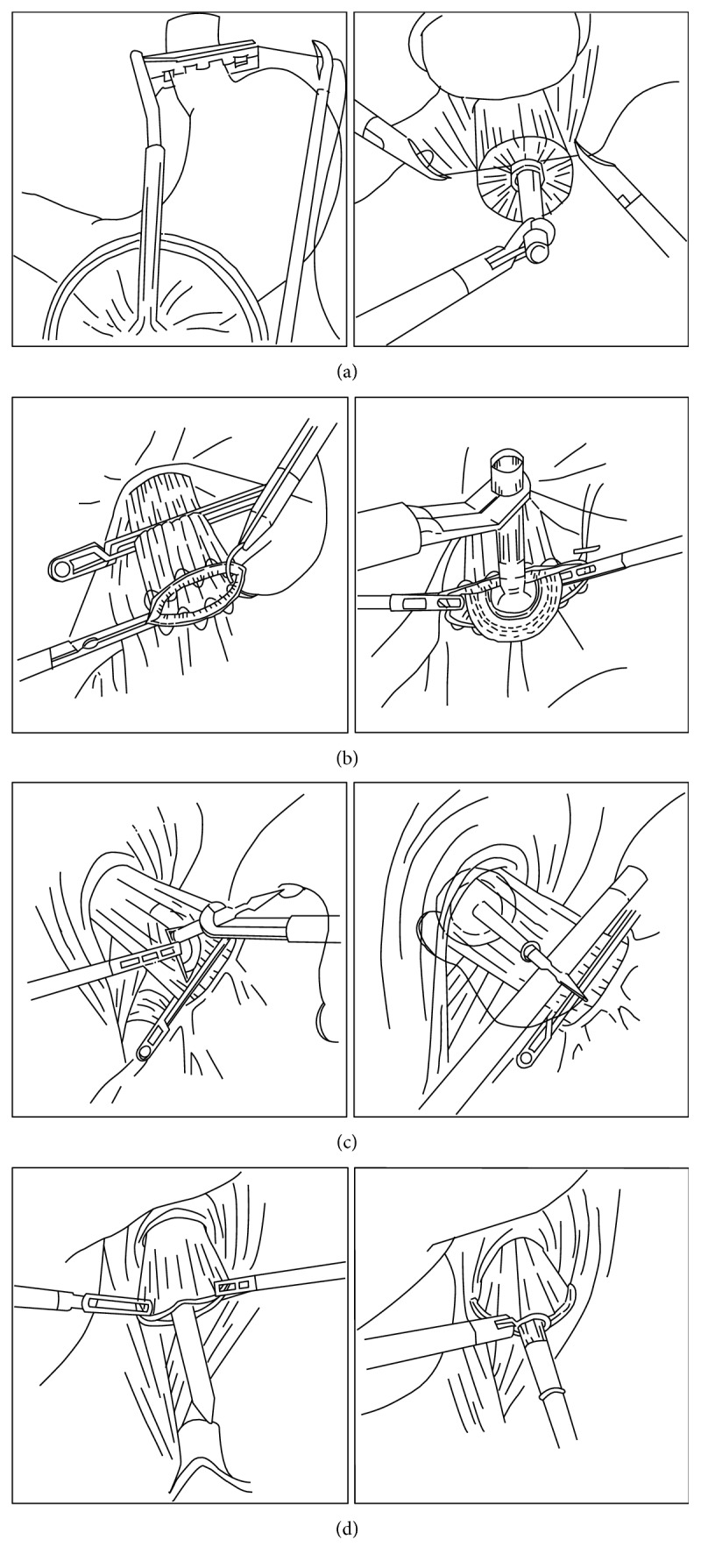
The several methods of anvil insertion for esophagojejunostomy using a circular stapler after total gastrectomy. (a) Anvil insertion using a small-head purse-string instrument. (b) Anvil insertion after laparoscopic hand-sewn purse-string sutures. (c) Insertion of an anvil attached with a thread through an esophagotomy at the anterior wall of the esophagus. (d) An oral anvil insertion method (OrVil).

Esophagojejunostomy using a circular stapler is the most famous technique in the open surgery. For extracorporeal approaches in LATG, a 4 to 6 cm-sized epigastric incision is made to access the esophagogastric junction. Many reports pertaining to the extracorporeal method describe the procedures in simple terms, such as “the insertion of an anvil with the purse-string suture.” However, the procedure is often very difficult because the purse-string device is too bulky to handle in a small minilaparotomy space, and suturing through a small incision in the deep and narrow operative field is an exacting process [31, 32]. However, a purse-string instrument with a smaller head has been developed, which can be inserted through a 12 mm port into the abdominal cavity. Accordingly, a purse-string suture can be performed under laparoscopic vision [33] (Figure 1(a)). Some surgeons prefer intracorporeal hand-sewn purse-string sutures because of their relative simplicity (Figure 1(b)). Secured anvil insertion into the esophageal stump is the next challenging step. After the purse-string suture, the esophageal stump is opened by two forceps, at which point, an anvil can be inserted easily [34, 35]. Another method for laparoscopic anvil insertion is to use an anvil attached with a thread through a small esophagotomy at the anterior wall of the esophagus. The thread is then pulled outside to place the anvil at the esophageal edge, and the esophagus is transected using a linear stapler [36] (Figure 1(c)). Oral insertion of an anvil (Orvil, OrVil™, Covidien, Tokyo, Japan) was introduced as an alternative to reduce procedure complexity [37, 38] (Figure 1(d)). Despite concerns, the esophageal mucosal injury, a specific complication of a transoral anvil passage, would occur; such mucosal injury has been rarely reported [39]. The location of the camera port and the small size of the incision for circular stapler insertion are also very important to enable a proper view during this procedure. Most surgeons prefer an upper midline incision for anvil insertion in cases of extracorporeal anastomosis. The same incision is used for the insertion of a circular stapler with a laparoscope from the left lower port [31] or the left upper port [40]. Some surgeons use an extended umbilical port wound [41] or a left lower port wound [37].

#### 2.4.4. Esophagojejunostomy Using Linear-Stapled Anastomosis

The handling of the linear stapler is easier in LTG field because linear staplers are thinner and have superior mobility of the tip compared to circular staplers. Moreover, surgeons can perform the linear-stapled anastomosis regardless of the esophageal diameter and can achieve a larger anastomosis than in the case of circular-stapled anastomosis. In addition, linear-stapled anastomosis helps the surgeon reduce the torsion of the jejunal limb, which is a severe complication of circular-stapled anastomosis.

**Figure 2:**
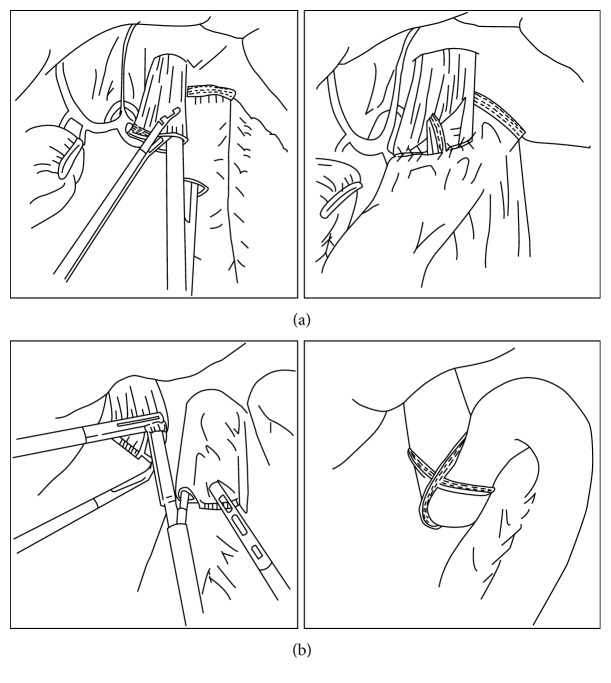
Two representative types of anastomosis using linear stapler after total gastrectomy. (a) The overlap method. (b) The functional end-to-end anastomosis method.

For the overlap method (Figure 2(a)), a small entry hole is made about 5 cm distal to the stapler line on the jejunal limb, while another enterotomy is made on the left wall of the esophageal stump. An anastomosis is performed by inserting the stapler between the esophageal enterotomy and the entry hole of the jejunal limb toward the cephalic side of the lumen. The jejunal limb is positioned at the left side of the esophageal stump. Then, the common entry hole is closed using an intracorporeal hand-sewn suture. For the functional end-to-end anastomosis (FEEA) (Figure 2(b)), the esophagus is transected intracorporeally in the horizontal direction. Two small entry holes are made at the edges of the tip of the jejunal end and the transected esophagus. The linear stapler is inserted into the holes, and the anastomosis is created. The common entry hole is closed with a linear stapler.

There are two differences between the overlap method and FEEA: the peristaltic direction of the esophagojejunostomy and the methods of closing the common entry hole. Esophagojejunostomy in FEEA is performed in the antiperistaltic direction; therefore, the jejunal limb needs to be lifted further up in FEEA than in the overlap method. In addition, the closure of the common entry hole in FEEA is performed using a linear stapler, whereas the closure is performed using an intracorporeal suture in the overlap method. Thus, since a larger working space is needed in FEEA than in the overlap method, the dissection around the crura of the diaphragm has to be performed in FEEA, which can increase the risk of a diaphragmatic hernia. In contrast, advanced suturing skills are needed in the overlap method, and intracorporeal suturing frequently results in prolonged operative time [42].

### 2.5. Laparoscopic Function-Preserving Surgery

#### 2.5.1. Pylorus-Preserving Gastrectomy (PPG)

PPG was first introduced for the treatment of peptic ulcer, and since then, it has been used as a surgical treatment for EGC to preserve function and maintain a better QOL [43, 44]. According to Japanese gastric cancer treatment guidelines, PPG is the treatment option for clinically T1N0M0 gastric cancers in the middle third of the stomach at least 4.0 cm away from the pylorus. The distance from the lesion to the pylorus needs to be carefully considered because a short antral cuff may lead to postoperative gastric stasis, which is one of the typical complications of PPG. Considering the need for a sufficient distal resection margin of >1 cm for EGC, in addition to the length of the antral cuff, the distance from the lesion to the pylorus should be greater than 4.0 cm. The standard technique for PPG includes preservation of the hepatic branch of the vagus nerve and the infrapyloric vessels to allow for the structural and functional preservation of the pylorus. The LND of station 5 is usually omitted during PPG to preserve the hepatic branch of the vagus nerve. Finally, gastrogastrostomy is performed using either the hand-sewn method or the linear stapler. By preserving pyloric function, it confers potential nutritional advantages and carries a lower incidence of disturbed bowel habits, as well as fewer postgastrectomy disorders such as dumping syndrome and alkaline reflux [45]. Recently, laparoscopy-assisted PPG (LAPPG) has been reported to be beneficial compared to conventional PPG in terms of the preservation of functionality and minimal invasiveness [46].

#### 2.5.2. Vagus Nerve-Preserving Gastrectomy (VPG)

VPG was designed to decrease postgastrectomy symptoms caused by injury to the vagus nerve. It preserves not only the celiac branches of the posterior vagal trunk that innervate the small intestine but also the hepatic branches of the anterior vagal trunk that innervate the liver and biliary tract [47]. However, there is a specific concern regarding the incomplete LND for nerve preservation. During the LND, nonvisible nerve injury can occur because of energy devices and traction, making it difficult to achieve the complete removal of lymph nodes without nerve injury. Even if the nerve is grossly preserved, it is hard to confirm whether the function of the nerve has been preserved. Furthermore, a longer operative time is required to dissect around the nerve compared to that of conventional gastrectomy. In spite of these technical difficulties, VPG continues to be performed using auxiliary methods such as nerve monitoring according to an ongoing study that has not been published.

#### 2.5.3. Proximal Gastrectomy (PG)

In laparoscopic PG (LPG), the type of reconstruction may be the most important issue. Esophagogastrostomy has served as the most simple reconstruction method, though most patients end up suffering from postoperative reflux esophagitis and/or anastomotic stricture. Antireflux procedures including fundoplication, gastric tube formation, pyloroplasty, and esophagopexy with crural repair have been attempted in order to reduce the incidence of anastomotic stricture and reflux esophagitis. However, the results of these efforts have been far from satisfactory [48–50], and two effective alternatives to esophagogastrostomy after PG have been introduced, namely, jejunal interposition and double-tract reconstruction (DTR). Jejunal interposition was introduced as a strategy to reduce severe reflux; however, laparoscopic jejunal interposition has not been frequently performed because of the inherent technical complexity of the creation of a jejunal flap and three anastomoses, with the consequently prolonged operative time [51, 52]. Alternatively, DTR consists of three anastomoses: Roux-en Y esophagojejunostomy, gastrojejunostomy 15 cm distant from the esophagojejunostomy, and jejunojejunostomy 20 cm distant from the gastrojejunostomy. Ahn et al. reported that LPG with DTR for proximal EGC showed excellent postoperative outcomes, particularly with respect to decreased reflux symptoms. This procedure also showed the tendency for improvements in nutritional status, acceptable oncologic outcome rates, surgical time, and complication rates [53]. This procedure involves the creation of an additional anastomosis, a gastrojejunostomy by stapling, which adds only 5–10 min to the conventional LTG anastomosis procedure. Lastly, delayed gastric emptying becomes less of a concern since even if delayed gastric emptying occurs, there is an alternative passage route for food, in contrast to jejunal interposition.

### 2.6. Single-Incision Distal Gastrectomy (SIDG)

Reduced port or single-port laparoscopic surgery was developed to reduce scars and surgical stress, and indeed, single-port laparoscopic surgery through the umbilicus does not leave any visible scar. A vertical 2.5 cm-sized transumbilical incision is made, and a commercial single-port device is placed in the umbilical incision. An additional assistant trocar is not needed. Then, a flexible scope is used to secure a clear view of the operative field. For effective dissection, curved instruments are used when operating in the lesser curvature side and the suprapancreatic area. Regarding suprapancreatic LND, the neck and body of the pancreas are sometimes overly protruded, which makes it difficult to dissect the lymph nodes behind the pancreas using a straight instrument from the umbilicus. SIDG is not yet generalized because of the continuing need for more advanced techniques and scarring evidences of oncological safety. In addition, although previous reports of SIDG showed comparable morbidity and an absence of open conversion, the risk of unexpected intraoperative accidents in pure, single-port surgery cannot be ignored. A large-scale study is necessary to confirm the safety and oncologic outcomes of SIDG.

### 2.7. Robot Gastrectomy

Robotic systems for surgery were implemented in early 2000 to overcome the drawbacks of laparoscopic surgery. The features of the robotic systems, such as 3-D vision, the elimination of physiologic tremor, and the articulated arms, assist the surgeon by facilitating manipulation in the surgical field [54]. An articulated endoscopic wrist allows the operator seven degrees of freedom, via 180° articulation and 540° rotation. Moreover, the magnified 3-D high-definition imaging system and very stable camera platform, which is controlled by the operator, are significant characteristics. Another advantage of robotic surgery involves its ability to enable more precise intracorporeal suturing, such that reconstruction after total gastrectomy is greatly facilitated [55]. With the use of wristed instruments, the meticulous dissection of cardiac LND, mobilization of the distal esophagus, and insertion of the anvil head into the esophageal stump are more easily performed. In addition, the learning curve of robotic surgery is quite steep because of its simplicity and the potential for early adaptation [56, 57]. Recently, the robotic surgery with image-guided assistance has been reported. Kim et al. [58] demonstrated that the operators could employ a vascular map and avoid vascular injury or damage to other organs through the intraoperative image-guidance feature. During robotic gastrectomy, the fine movements and magnified view allow surgeons to perform clear LND without any vascular injury and with minimal intraoperative bleeding and to preserve the small branches of the splenic vessels.

## 3. Clinical Outcomes

### 3.1. Morbidity and Mortality

In LDG, the incidence of operative complications is reportedly lower overall than that in conventional ODG. In a previous prospective RCT, we reported that LADG confers the clear advantage of fewer pulmonary complications as compared to open gastrectomy [59]. Furthermore, a meta-analysis of 5 RCTs and 17 non-RCTs with 3411 patients reported that LADG might result in less blood loss, less consumption of analgesics, and shorter hospital stays, without an increase in total hospitalization costs [60]. While Kim et al. (COACT 0301) [61] reported significant reductions in mild complications in LADG (LADG versus ODG; 23.2% versus 41.5%, *p* = 0.012), the Korean laparoscopic gastrointestinal surgery study (KLASS) group performed a phase 3 multicenter RCT (KLASS-01) to establish even stronger evidence, and their morbidity and mortality data was recently reported [62]. The overall complication rate was significantly lower in the LADG group (LADG versus ODG; 13.0% versus 19.9%, *p* = 0.001); in particular, the wound complication rate in the LADG group was significantly lower than that in the ODG group (3.1% versus 7.7%, *p* < 0.001). However, there were no significant differences in terms of either major intra-abdominal complications (7.6% versus 10.3%, *p* = 0.095) or mortality rates (0.6% versus 0.3%, *p* = 0.687) between the two groups. For cases of advanced cancer, a Chinese group recently reported the surgical safety of LDG with D2 LND when compared with conventional ODG [63]. In their multicenter prospective RCT, no significant difference in postoperative morbidity was shown (LDG versus ODG; 15.2% versus 12.9%, *p* = 0.285). The mortality rate was also similar between the two groups (LDG versus ODG; 0.4% versus 0%, *p* = 0.249).

In contrast, only a few studies have reported on the feasibility and safety of LTG and the results remain controversial [40, 64–66]. Some authors reported that LATG offers technical feasibility and safety, comparable to open total gastrectomy. LATG was also shown to be associated with fewer postoperative complications, less pain, and rapid recovery. However, the heterogeneity of the results may have been caused by the following limitations: the mostly retrospective nature of the studies, small sample sizes, mostly EGC cases, and short follow-up periods.

Recently, several retrospective studies reported that TLDG was technically feasible, less invasive, and safer than LADG [67–70]. In a meta-analysis including five studies with 652 patients, TLDG was associated with less intraoperative blood loss, earlier first flatus, and lower postoperative morbidity than LADG. Our group performed a prospective RCT to evaluate the overall feasibility of TLDG [71] in which it was shown that TLDG is as safe and feasible as LADG, with a comparable rate of complications. Indeed, short of our expectation, no significant differences were shown between the TLDG and LADG groups in terms of postoperative course such as recovery, postoperative pulmonary function, and inflammatory parameters. It could be the reason that the parameters used in the clinical field to evaluate the early surgical outcomes could not accurately reflect the subtle difference in surgical invasiveness between TLDG and LADG.

Recently, the Korean Robot Gastrectomy Study Group reported the results of a multicenter prospective, clinical trial comparing robotic gastrectomy with laparoscopic gastrectomy in EGC [72]. Patients were enrolled for treatment with either robotic (*n* = 185) or laparoscopic (*n* = 185) gastrectomy. The study showed similar overall complication rates (11.9% versus 10.3%; robotic versus laparoscopic) and major complication rates (1.1% versus 1.1%; robotic versus laparoscopic) with no operative mortality in either groups. In the robotic surgery group, the operative time was significantly longer (221 minutes versus 178 minutes; robotic versus laparoscopic, *p* < 0.001) compared with that in the laparoscopic group. There were no significant differences between the two groups regarding the rates of open conversion, operative blood loss, length of stay, or diet build-up. The authors concluded that robotic gastrectomy was not superior to laparoscopic gastrectomy. It is true that the robot group paid higher costs [South Korean won 13,748,422.5 (US$13,470) (robotic) versus 9,165,862 (US$8980) (laparoscopic); *P* < 0.001], but most of the high cost will not be a big problem in the future due to depreciation and maintenance costs. Rather than simply calculating the cost per operation, we should study the indications that require robotic gastrectomy, apply robotic surgery where necessary, and reduce the social burden by reducing complications.

### 3.2. Survival and Recurrence

Regarding oncological safety, our group reported the long-term safety of LADG for EGC [73]. Tumor recurrence occurred in 0.9% and the rate of cancer-related death was 0.5% (only one patient) during the median 55-month follow-up period, and the overall 5-year survival rate was not significantly different between the LADG and ODG groups (95.9% versus 94.9%; LADG versus ODG). Zeng et al. [60] also reported comparable long-term survival rates between both groups in a meta-analysis. The long-term results of a prospective RCT (COACT 0301) with 164 patients of the median 74.3-month follow-up period showed that the survival rates of the LADG and ODG groups were comparable (5-year overall survival: 97.6% versus 96.3, *p* = 0.721; 5-year disease-free survival: 98.8% versus 97.6% in LADG versus ODG, *p* = 0.514) [61].

There have been several studies on the oncologic outcomes of laparoscopic gastrectomy with extended lymphadenectomy for advanced gastric cancer (AGC). The findings of these studies suggested that the 3-year overall survival (75.3%) and disease-free survival (69.0%) for laparoscopic surgery were comparable to those of published studies on conventional open surgery [74–76].

Two multicenter RCTs, the KLASS-01 study and the Japanese Clinical Oncology Group (JCOG) 0912 trials, are currently ongoing on large-scale studies and seek to elucidate the oncological outcomes of laparoscopic gastrectomy for EGC. The KLASS-01 trial is the first multicenter RCT to compare laparoscopic surgery with open surgery in patients with clinical stage I gastric cancer. Overall survival is the primary endpoint, and the secondary endpoints include morbidity and mortality, disease-free survival, quality of life, inflammatory responses, and cost effectiveness. A total of 1416 patients (705 and 711 patients in the LADG and ODG groups, resp.) were enrolled from 2006 to 2010, and the final results will be reported in the near future [77]. The JCOG started a multicenter RCT in 2010 to compare LADG with ODG in 920 patients with stage I gastric cancer from 33 institutions. In the JCOG 0912 study, the overall survival rate is the primary endpoint, and the secondary endpoints include short-term clinical outcomes, adverse events, proportion of LADG completion and conversion to open surgery, relapse-free survival, and postoperative QOL [78].

The long-term results of laparoscopic surgery for AGC remain controversial because of the paucity of reliable studies. While D2 LND is the standard for AGC treatment in open, laparoscopic, and robotic surgeries, it is very challenging to perform. Even in East Asia, where there are many more cases and experiences of gastric cancer than in Western countries, only a few qualified surgeons are capable of performing a complete D2 LND using a laparoscopic approach. Significant improvements in laparoscopic surgical instruments and techniques have been progressing for decades [79]. This accumulation of laparoscopic expertise has led to the use of extended LND and to attempts by some experienced surgeons to extend the indication of laparoscopic gastrectomy to locally advanced cases. Recently, it was reported that no significant difference in overall survival and disease-free survival between open gastrectomy and laparoscopic gastrectomy in AGC was shown in a meta-analysis using 10 studies with a total of 1819 patients [80]. There are several difficulties or limitations in applying laparoscopic surgery to AGC, including total omentectomy, splenic hilar dissection for proximal gastric cancer, bulky positive nodes or large primary tumor, esophageal invasion, and peritoneal lavage [81]. Several data reported that laparoscopic gastrectomy with D2 LND was technically feasible and safe for patients with AGC, with acceptable rates of morbidity and mortality and satisfactory long-term outcomes [74–76, 82, 83]. Achieving excellent long-term results in laparoscopic gastrectomy depends on the standardization of the D2 LND. To that end, the KLASS team sought to achieve a consensus as to the D2 LND procedure [84]. All surgeons participating in the KLASS study were each asked to submit three laparoscopic and three open D2 gastrectomy videos. Each unedited video was allocated to several peer reviewers and reviewed blindly. Based on the results of experts' reviews, the review evaluation committee decided whether the surgeon could be included in the KLASS-02 trial. This systematic approach will serve as a crucial example for surgical standardization.

Three multicenter trials, the KLASS-02, the Japanese Laparoscopic Surgery Study Group (JLSSG) 0901, and the CLASS (Chinese laparoscopic gastrointestinal surgery study)-01, are current large-scale studies seeking to elucidate the oncological outcomes of laparoscopic gastrectomy for AGC. The KLASS-02 trial is a phase III study evaluating the efficacy of LDG with D2 LND for AGC. The primary endpoint is 3-year disease-free survival, and the estimated population size is 1050. The JLSSG 0901 trial is a phase II/III study comparing LADG and ODG in patients with clinical T2 to T4aM0 gastric cancer. After the recruitment of 180 patients, the rate of major complications will be analyzed. The study will continue the recruitment until 500 patients are enrolled unless there is early termination due to a high complication rate [85]. Lastly, the CLASS-01 trial performed by the Chinese group is a phase III study, and the study design is similar to that of the KLASS-02 trial. In the near future, these well-designed studies may help to establish concrete, reliable evidence for the expansion of the indication for laparoscopic gastrectomy to advanced cases.

### 3.3. Quality of Life

Laparoscopic gastrectomy has many merits over open gastrectomy, such as less pain, shorter postoperative hospital stays, earlier recovery, and superior cosmetic outcomes. In several meta-analyses, the superiority of laparoscopic gastrectomy in terms of postoperative recovery was shown [86, 87]. In another report, LADG showed improved short-term symptoms and functional outcomes [61].

Function-preserving surgery was introduced to improve the QOL of patients. Recently, the nutritional and functional benefits of LAPPG were compared to those of LADG [46]. It was reported that the incidence of delayed gastric emptying was lower, though other complications occurred more frequently in LADG than in LAPPG. Decreased serum albumin and protein levels at one to six months postoperatively and greater abdominal fat volumes at postoperative one year were observed in LADG compared with LAPPG. The authors concluded that LAPPG could be considered a superior treatment option for middle-third EGC over LADG in terms of its lower incidence of gallstone and nutritional advantages. In addition, an RCT reported that the patients who underwent VPG showed significantly less diarrhea and less appetite loss at 12 months. They concluded that VPG could improve postoperative QOL compared with conventional gastrectomy [47].

In proximal EGC, the application of PG has been limited until now. In a systematic meta-analysis comparing TG with PG, PG with esophagogastrostomy showed a higher incidence of reflux esophagitis and anastomotic stenosis [88]. However, several positive results of LPG with modified reconstruction methods have been reported [48, 89]. Accordingly, LPG can be considered an attractive treatment option for proximal EGC as a minimally invasive surgery to preserve functionality, including reduction of postoperative complaints, prevention of anemia, improved nutrition, and improved production of gut hormones [90–93].

## 4. Conclusions

Laparoscopic gastrectomy has advanced to look for minimally invasive approaches as well as to maintain the oncologic safety. In accordance with the evolution of surgical instrumentation and increased laparoscopic surgical experience, its indication has been extended to advanced cases. Recent studies show that the oncologic outcomes of laparoscopic gastrectomy for EGC are comparable to those of open gastrectomy. The demonstration of a similarly optimal result regarding the safety of laparoscopic gastrectomy in AGC is awaited. The results of several ongoing multicenter RCTs are expected to establish concrete evidence of the widespread suitability of laparoscopic gastrectomy in the treatment of gastric cancer.

## Figures and Tables

**Table 1:** Previous historical report of laparoscopic gastrectomy for gastric cancer.

Author	Year	Operation	Indication	Country	Analysis
Ohgami et al.	1992	Laparoscopic wedge resection	EGC	Japan	*n* = 6
Ohashi et al.	1995	Laparoscopic intragastric mucosal resection	EGC	Japan	*n* = 8
Kitano et al.	1994	Laparoscopic-assisted Billroth I gastrectomy	EGC	Japan	Case report
Azagra et al.	1999	Laparoscopic-assisted total gastrectomy	EGC, AGC	Belgium	*n* = 13
Uyama et al.	1999	LADG with D2 LND	AGC	Japan	*n* = 12
Ohki et al.	1999	Hand-assisted laparoscopic distal gastrectomy	EGC	Japan	Case report
Kanaya et al.	2002	Totally laparoscopic Billroth I gastrecotmy	EGC	Japan	*n* = 9
Giulianotti et al.	2003	Distal and total robotic gastrectomy	EGC, AGC	Italy	*n* = 18
Omori et al.	2011	Single-incision laparoscopic distal gastrectomy	EGC	Japan	*n* = 7

## Additional Points

*Core Tip*. Laparoscopic gastrectomy will continue to advance toward increasingly minimally invasive approaches to improve the quality of life of patients with gastric cancer, without compromising oncologic safety.
